# Knowledge, Attitude, Awareness, and Future Expectations of Robotic Surgery in Patients Attending Surgical Specialties Clinics

**DOI:** 10.7759/cureus.56523

**Published:** 2024-03-20

**Authors:** Fahad A Al Dihan, Mohannad A Alghamdi, Faisal A Aldihan, Nawaf M Alamer, Faisal A Alshahrani, Ayyob Alqarni

**Affiliations:** 1 College of Medicine, King Saud Bin Abdulaziz University for Health Sciences, Riyadh, SAU; 2 General and Colorectal Surgery, King Abdulaziz Medical City Riyadh, Riyadh, SAU

**Keywords:** patients' perception, minimally invasive surgery, survey, public perspectives, robotic-assisted surgery

## Abstract

Introduction

The use of robotic-assisted surgeries (RAS) has been growing in surgical specialties. It allows surgeons to perform higher-quality operations with fewer complications, mortality, and morbidity. However, there are a lot of misconceptions about RAS among patients. Therefore, our study aimed to assess the knowledge, attitude, awareness, and future expectations of RAS in patients attending surgical clinics.

Methods

A cross-sectional study was conducted in King Abdulaziz Medical City (KAMC) surgical clinics in Riyadh, Saudi Arabia. All participants <18 years of age were excluded. The questionnaire was distributed to 304 patients attending surgical clinics with a confidence level of 95% and a margin of error of 5%. Cluster sampling was used since the respondents were from multiple surgical specialties. Finally, multivariate analysis was performed to assess participants' preference for robotic surgery.

Results

Most participants (58.6%, n=178) were between 21 and 40 years old, and males were 52% of the participants. Many respondents thought a robot did not do the surgery. 70.7% of respondents had not heard of robotic surgery, with the media being the most common source of information. Internal damage was the prevalent concern (51.0%, n= 155) in malfunctions of robotic surgery. A significant relationship was found between participants from 21 to 40 years of age and a stronger preference for robotic surgery (p=.027). Respondents who preferred robotic surgery were discovered to have a significant relationship with participants who thought robotic surgery was safer and had better results (p<.001). 13.9% of participants who did not prefer robotic surgery also took cost into account significantly (χ2=28.93, p<.001, Cramer's V=.22). 67.2% (n=43) of respondents who preferred robotic surgery believed it might eventually replace present practices.

Conclusion

Our study concluded that the majority did not favor or were unsure whether to undergo robotic surgeries or not. However, most participants had some misconceptions and a lack of awareness about robotic surgeries. Raising awareness among patients can improve the mutual decision-making between them and their treating physician.

## Introduction

A major leap in the field of surgery was the introduction of minimally invasive surgery (MIS) through advancements in robotics and medicine. These advancements led to the first laparoscopic surgery, performed in 1983 by the German gynecologist Karl Semm. Since then, surgeons have begun shifting from performing traditional surgery to MIS. The adoption was initially slow due to skepticism, but MIS proved to be better due to improvements in the length of hospitalization, faster recovery times, and lower risk of infections. Laparoscopic procedures are the standard for multiple surgeries and the most common form of MIS [1,2]. However, the new cutting-edge robotic systems provide much more advanced technologies, such as more stability, improved visualization, and elimination of the fulcrum effect (seen in laparoscopic surgery) [3].

Since the advent of robotic surgery, several surgical specialties embraced it. Robotic-assisted surgeries (RAS) have been growing, especially in cardiothoracic, head and neck, urology, gynecology, and general surgeries [4]. A study showed that in colorectal RAS, there was a reduction in conversion to open surgery and similar perioperative outcomes compared to a laparoscopic colectomy [5]. However, cost and longer operation time disadvantaged many hospitals [6]. According to a study, the complication rate of robotic-assisted breast reconstruction surgeries was 16.7% compared to open breast surgeries, which was 37.5%. Also, in the study, the results of RAS satisfied cosmetic and patient esthetic concerns, but its cost was an issue [7]. Robotic surgeries have also been applied to head and neck surgeries. Robotic-assisted thyroid surgery showed accurate dissection and a more magnified view [8]. In head and neck RS, tumor excision exhibited comparable oncologic outcomes to the standard surgical procedure [9]. 

In thoracic surgery, robotics has made huge advancments in lung cancer and mediastinal surgeries since it improved the mean operative time, mean length of stay, mortality, and morbidity rates [10]. Also, an international retrospective study on stage III lung cancer showed excellent oncological outcomes and postoperative results, which proved safe [11]. Moreover, the 3D magnified view in robotic anterior mediastinum surgery allowed the application of different maneuvers in tight spaces [10]. In urology, the da Vinci surgical system (Intuitive Surgical, Sunnyvale, California) is used for surgical procedures such as robotic‐assisted prostatectomy. Since then, there has been an increase in the number of robotic procedures performed in the past five years, from 1500 performed procedures in 2000 to more than 20,000 in 2004 [12]. Endometrial cancer, compared to conventional laparoscopic, had better wound complication rates and less blood loss. Similarly, patients who underwent robotic-assisted surgery for ovarian cancer had less blood loss and a shorter hospital stay [13].

RAS allows surgeons to perform higher-quality operations with less blood loss, complications, mortality, and morbidity compared to laparoscopic or open surgeries [14]. However, RAS usually have longer operation times and are more expensive [15]. Moreover, with the growth of RS in practice, there are not many studies in the literature that assessed patients' perceptions and attitudes toward RS. One study that was conducted on the general population in the United States stated that 86% of the 747 participants had heard of robotic surgery, but almost 25% had a misconception that robotic surgery is open, laser, or scarless surgery [16]. Another study in Singapore showed that 53% of the 472 patients and their relatives from the outpatient clinic have heard of robotic surgery, with 82% of the participants being introduced to it by social media. Moreover, 43% of the participants had a misconception that an automated robot is involved in robotic surgery [1].

No local study has assessed the general publics' or patients' knowledge of robotic surgeries. Furthermore, the primary aim of this study is to assess the attitude, knowledge, awareness, and future expectations of patients attending specific surgical outpatient clinics, including thoracic surgery, colorectal surgery, urology, and gynecology clinics. Another aim was to also compare the results with other similar global studies.

## Materials and methods

This cross-sectional study will be conducted in King Abdulaziz Medical City (KAMC) surgical clinics in Riyadh, Saudi Arabia, to assess the patient's or their companion's/relatives' knowledge, attitude, awareness, and future expectations of robotic surgery. The surgical clinics included are obstetrics and gynecology (OBGYNE), thoracic, urology, and colorectal surgery. The choice of the clinic was due to the fact that the available robotic surgeries in KAMC are under those specialties. The inclusion criteria will be those who are above the age of 18, Saudis and non-Saudis, and both genders. The questionnaire with the consent will be distributed to all patients attending surgical clinics, 385 participants, with a confidence level of 95% and a margin of error of 5%. Finally, the sampling technique will be Cluster sampling since the respondents will be from multiple surgical specialties.

Survey description

The survey was developed based on the survey of Boys et al. [16]. There was no Arabic version, thus, backward and forward translation processes were applied to construct an Arabic version of the survey. The original English version was sent to a professional translator to translate it into Arabic. Then, another professional translator translated the Arabic version into English. Finally, both versions were compared, and there was no difference between the original and final English versions. The survey was constituted of two sections. The first included patient demographics, such as age, gender, education level, and nationality. The second section asked questions assessing the patient's perception/knowledge, awareness, attitude, and future expectations of robotic surgery.

Study protocol

The study's participants were selected by convenience sampling. Patients or their relatives waiting for their appointments at the outpatient surgical clinics were approached to participate in the study or if they preferred not to (those who refused to participate were not contacted further). Additionally, the patients were told that participation would not require personal information such as their name, contact information, or any identity document (hospital card, government documents, etc.). The participants were told that the survey would take around 10 minutes to finish. Also, they were informed that at any point in time, for whatever reason, participants can withdraw at will without any consequences. Before handing the survey to the participants, the purpose of the study and the components of the survey were explained to them, and they were told to read the consent and agree to it. Once the patients consented to participate in the study, they were given the survey to finish, and they were instructed to put the finished survey when leaving the outpatient clinics in a designated box.

Data analysis

The data collected was registered and analyzed using the Statistical Package for the Social Sciences (SPSS) version 25 (IBM Inc., Armonk, New York). Percentages and frequencies present categorical variables such as the level of education, while mean and standard deviation will present quantitative variables like age. A p-value less than 0.05 was considered statistically significant. The Chi-squared test (χ2) assessed the statistical association between sociodemographic factors, attitudes, perceptions, and future expectations and surgical preference among participants who either preferred robotic surgery, did not prefer robotic surgery, or were unsure of their preference. Cramer's V was used to analyze the strength of the association; a value of <0.20 denotes a weak association, 0.20-0.30 is a moderate association, and >0.30 is a high association.

## Results

In this study, 304 participants were recruited and completed the survey. Most participants (58.6%, n=178) were between 21 and 40 years old. Regarding gender, 52% of the participants were male. Only 2.6% (n=8) of the participants were non-Saudi, while the remaining 97.4% (n=296) were Saudi. Secondary school made the largest category in terms of education, accounting for 37.5% (n=114) of participants, followed by participants with an undergraduate degree (32.6%, n=99). Most participants attended the OBGYNE (29.9%, n=91) clinics, followed by urology clinics (29.6%, n=90). Most participants (76.3%, n=232) had undergone or knew a family member who had surgery. Moreover, most respondents said a robot did not do the surgery (91.4%, n=278; Table 1).

**Table 1:** Sociodemographic profile of participants

Characteristics	n=304 (%)
Age	
21-40	178 (58.6)
41-60	90 (29.6)
61-80	29 (9.5)
>80	7 (2.3)
Gender	
Male	158 (52.0)
Female	146 (48.0)
Nationality	
Saudi	296 (97.4)
Non-Saudi	8 (2.6)
Highest educational level	
Primary school	32 (10.5)
Secondary school	114 (37.5)
Diploma	44 (14.5)
Undergraduate degree	99 (32.6)
Postgraduate degree	15 (4.9)
Surgical clinic attended	
Colorectal surgery	70 (23.0)
OBGYNE	91 (29.9)
Thoracic surgery	53 (17.4)
Urology	90 (29.6)
Has a surgical procedure been performed on yourself, a family member, or friends?	
Yes	232 (76.3)
No	72 (23.7)
Was it by robotic surgery?	
Yes	26 (8.6)
No	278 (91.4)

In terms of awareness, around two-thirds of the participants (70.7%, n=215) had not heard of robotic surgery, with the media being the most prevalent source of information (63.8%, n=87), followed by friends and relatives (20%, n=20). Regarding what kind of operation robotic surgery most resembles, 47.7% (n=145) said laparoscopic surgery. When asked about their understanding of robotic surgery, the majority (34.5%, n=105) said that the surgeon controls the robot to perform the surgery, while a significant portion (33.6%, n=102) believed that a robot performs the surgery while a trained surgeon observes the robot. In terms of awareness, around two-thirds of the participants (70.7%, n=215) had not heard of robotic surgery, with the media being the most prevalent source of information (63.8%, n=87), followed by friends and relatives (20%, n=20). Most participants (47.7%, n=145) thought that laparoscopic surgery resembles robotic surgery the most. When asked about their understanding of robotic surgery, the majority (34.5%, n=105) said that the surgeon controls the robot to perform the surgery, while a significant portion (33.6%, n=102) believed that a robot performs the surgery while a trained surgeon observes the robot. When participants were asked to compare robotic surgery to traditional surgery, their opinions varied. Some believed robotic surgery was safer (22.0%, n=67), faster (18.4%, n=56), and producing better results (10.5%, n=32). Only a small fraction felt it was less painful or expensive than traditional surgery (8.2%, n=25, each), while a significant number of people (32.6%, n=9) did not see any benefits. Most participants had concerns about robotic surgery, including robot malfunctions causing internal damage (51.0%, n=155) and the robot executing the incorrect procedure (31.3%, n=95). Meanwhile, only (12.5%, n=38) of respondents thought surgical robots had never malfunctioned, while the majority (83.6%, n=254) were unsure. Regarding attitude, 43.4% (n=132) of participants were unsure if they would prefer to undergo robotic surgery, while 35.5% (n=108) preferred not. Considering participants' perceptions of surgeons trained in robotic surgery, 35.5% (n=108) believed they were more competent. Regarding hospitals utilizing robotic surgery, 61.8% (n=188) of respondents thought they were better. Compared to traditional laparoscopic surgery, 48% (n=146) thought they were more expensive. When asked if robotic surgery could replace conventional surgical procedures, 40.8% (n=124) of respondents were unsure. Similarly, 48.6% (n=148) of respondents were unsure whether robotics could improve surgical results (Table 2).

**Table 2:** Awareness, perception, knowledge, attitude, and future expectations of the participants toward robotic surgery *Frequencies do not add to 304 due to multiple option selection

Characteristics	n=304 (%)
Awareness	
Heard about robotic surgery	
Yes	89 (29.3)
No	215 (70.7)
Source of information*	
Media	87 (63.8)
Magazine	7 (5.2)
Doctor	15 (11.0)
Friends and relatives	27 (20.0)
Perception and/or knowledge	
Which type of surgery is robotic surgery most similar to?	
Traditional open surgery	27 (8.9)
Laparoscopic surgery	145 (47.7)
Laser surgery	104 (34.2)
Don't know or missing response	28 (9.1)
Understanding of robotic surgery	
Robot performs surgery, trained surgeon stands by	102 (33.6)
Surgeon controls robotic arms and instrument	105 (34.5)
Surgeon tells robot what to do, robot follows each command	43 (14.1)
Surgeon not present in the operating theatre; robot performs according to software	28 (9.2)
Unsure/ don't know/ NA	26 (8.6)
Perceptions of robotic surgery compared to non-robotic surgery	
Faster	56 (18.4)
Safer	67 (22.0)
Better results	32 (10.5)
Less painful	25 (8.2)
Costly	25 (8.2)
None of the above	99 (32.6)
Concerns regarding robotic surgery	
Robot malfunctions causing internal damage	155 (51.0)
Robot performs wrong procedure	95 (31.3)
Unsure (NA)	54 (17.7)
Do you think robotic malfunction has occurred before during surgery?	
Never	38 (12.5)
Yes	12 (3.9)
Unsure	254 (83.6)
Attitude	
Would you prefer to undergo robotic surgery?	
Yes	64 (21.1)
No	108 (35.5)
Unsure	132 (43.4)
Perceptions of surgeons trained in robotic surgery	
More skilled	108 (35.5)
Similar	101 (33.2)
Less skilled	61 (20.1)
Unsure	34 (11.2)
Perceptions of hospitals using robotic surgery	
Better	188 (61.8)
Similar	78 (25.7)
Worse	8 (2.6)
Unsure	30 (9.8)
Compared to standard laparoscopic surgery, robotic procedures cost	
Less	62 (20.4)
Same	59 (19.4)
More	146 (48.0)
Unsure	37 (12.2)
Future expectations	
Robotic surgery can replace currently used surgical procedures	
Yes	106 (34.9)
No	74 (24.3)
Unsure	124 (40.8)
The use of robotics in surgeries can improve surgical outcomes	
Yes	116 (38.2)
No	40 (13.2)
Unsure	148 (48.6)

A significant relationship between age and surgical choice was found (χ2=10.29, p=.027, Cramer's V=.13), with participants aged 21 to 40 showing a stronger preference for robotic surgery (46.9%, n=30). When perceptions of robotic surgery were compared to perceptions of non-robotic surgery, significant variations were found (χ2=72.98, p<.001, Cramer's V=.35). Those who preferred robotic surgery thought it to be safer (48.4%, n=31) and having better results (20.3%, n=13). Also, cost was considered by 13.9% (n=15) of those who did not prefer robotic surgery. The opinions of surgeons with robotic surgical training differed significantly (χ2=28.93, p<.001, Cramer's V=.22).

Participants, including those who did not prefer robotic surgery (35.2%, n=38), did prefer it (45.3%, n=29), and were undecided about their opinion (31.1%, n=41), thought that surgeons with robotic surgery training had higher levels of skill. Significant differences were found in how respondents perceive hospitals that use robotic surgery (χ2=35.98, p<.001, Cramer's V=.24). Regardless of the preferred surgical method; participants believed that hospitals adopting robotic surgery were better (61.8%, n=188). Robotic surgery preference and the idea that it can replace current practices had a strong association (χ2=59.34, p<.001, Cramer's V=.31). 67.2% (n=43) of respondents who preferred robotic surgery thought it may eventually replace present practices. The opinion that robotic surgery can improve surgical outcomes also showed a significant link (χ2=91.86, p<.001, Cramer's V=.39). 78.1% (n=50) of those who preferred robotic surgery thought it could improve surgical outcomes (Table 3).

**Table 3:** Sociodemographic, attitudes, perceptions, and future expectations of participants of those who preferred robotic surgery, those who did not, and those who were unsure

	Total (n=304)	Does not prefer robotic surgery (n=108)	Prefers robotic surgery (n=64)	Unsure about their preference (n=132)	Univariate analysis		
					χ^2^	p-value	Cramer's V
Age							
21-40 years	178 (58.6)	75 (69.4)	30 (46.9)	73 (55.3)	10.29	.027	.13
41-60 years	90 (29.6)	21 (19.4)	24 (37.5)	45 (34.1)			
Above 60	36 (11.8)	12 (11.1)	10 (15.6)	14 (10.6)			
Gender							
Female	146 (48.0)	59 (54.6)	23 (35.9)	64 (48.5)	5.65	.059	.14
Male	158 (52.0)	49 (45.4)	41 (64.1)	68 (51.5)			
Education level							
Primary	32 (10.5)	8 (7.4)	9 (14.1)	15 (11.4)	4.51	.808	.09
High school	114 (37.5)	43 (39.8)	22 (34.4)	49 (37.1)			
Diploma	44 (14.5)	14 (13.0)	7 (10.9)	23 (17.4)			
Undergraduate	99 (32.6)	37 (34.3)	23 (35.9)	39 (29.5)			
Postgraduate	15 (4.9)	6 (5.6)	3 (4.7)	6 (4.5)			
Perceptions of robotic surgery compared to non-robotic surgery							
Faster	56 (18.4)	19 (17.6)	12 (18.8)	25 (18.9)	72.98	< .001	.35
Safer	67 (22.0)	7 (6.5)	31 (48.4)	29 (22.0)			
Better results	32 (10.5)	5 (4.6)	13 (20.3)	14 (10.6)			
Less painful	25 (8.2)	12 (11.1)	4 (6.3)	9 (6.8)			
Costly	25 (8.2)	15 (13.9)	1 (1.6)	9 (6.8)			
None of the above	99 (32.6)	50 (46.3)	3 (4.7)	46 (34.8)			
Concerns regarding robotic surgery							
Robot malfunctions causing internal damage	155 (51.0)	63 (58.3)	32 (50.0)	60 (45.5)	6.95	.139	.11
Robot performs wrong procedure	95 (31.3)	31 (28.7)	23 (35.9)	41 (31.1)			
NA	54 (17.8)	14 (13.0)	9 (14.1)	31 (23.5)			
Perceptions of surgeons trained in robotic surgery							
More skilled	108 (35.5)	38 (35.2)	29 (45.3)	41 (31.1)	28.93	< .001	.22
Similar	101 (33.2)	27 (25.0)	29 (45.3)	45 (34.1)			
Less skilled	61 (20.1)	33 (30.6)	5 (7.8)	23 (17.4)			
NA	34 (11.2)	10 (9.3)	1 (1.6)	23 (17.4)			
Perceptions of hospitals using robotic surgery							
Better	188 (61.8)	55 (50.9)	55 (85.9)	78 (59.1)	35.98	< .001	.24
Similar	78 (25.7)	43 (39.8)	6 (9.4)	29 (22.2)			
Worse	8 (2.6)	4 (3.7)	1 (1.6)	3 (2.3)			
NA	30 (9.9)	6 (5.6)	2 (3.1)	22 (16.7)			
Future expectations							
Robotic surgery can replace currently used surgical procedures							
Yes	106 (34.9)	24 (22.2)	43 (67.2)	39 (29.5)	59.34	< .001	.31
No	74 (24.3)	45 (41.7)	8 (12.2)	21 (15.9)			
Unsure	124 (40.8)	39 (36.1)	13 (20.3)	72 (54.5)			
Robotics in surgeries can improve surgical outcomes							
Yes	116 (38.2)	22 (20.4)	50 (78.1)	44 (33.3)	91.86	< .001	.39
No	40 (13.2)	30 (27.8)	7 (10.9)	3 (2.3)			
Unsure	148 (48.7)	56 (51.9)	7 (10.9)	85 (64.4)			

## Discussion

In the last decades, the role of robotic surgery has become imminent with advances in the surgical field. Robotic surgery has proved to be a better option in a lot of cases compared to traditional procedures since it causes fewer complications. Patients in this day and age should understand the advances in surgery and be informed of the operation they are going through [17]. Thus, our study aimed to assess the attitude, knowledge, awareness, and future expectations of patients attending surgical outpatient clinics in KAMC. Even though the number of RAS in Saudi Arabia has increased more than ever before, the findings illustrate the lack of knowledge regarding RAS [18, 19]. Understanding the types of procedures is vital in the current era of modern medicine since patients must make informed decisions based on their understanding of the operation they will undergo.

The majority of the participants believed that laparoscopic surgery (47.7%) is closest to robotic surgery, which is similar to other studies by Boys et al. (78.0%) and Kai et al. (64.4%) [16, 1]. Regarding the misconception of robotic autonomy, our study demonstrated higher percentages (56.9%) compared to Boys et al. (21%) and Kai et al. (43.6%) [16, 1]. Similarly, the study that was done by Irani et al. showed that most of the respondents (67.5%) didn't know that the robot was controlled manually by the surgeon [20]. Regarding participant's perception of the comparison between robotic and non-robotic surgeries, only a minority of participants believed robotic surgery to be safer (22%), less painful (8.2%), and to have a better outcome (10.5%), which is similar to the findings demonstrated by Kai et al. (29.2%, 14%, and 29%, respectively) [1]. In contrast, the study by Boys et al. showed a better perception of robotic surgeries in which most participants (72.0%) believed robotic surgery was safer, less painful, and/or offered better outcomes [16]. Moreover, our study showed almost half of our participants (51%) had concerns about malfunctions that would cause internal damage, which is less than both studies by Boys et al. (67.0%) and Kai et al. (79.0%) [16, 1].

A low rate of favorability for robotic surgery was observed among participants, with 35.5% dissenting it while 43.4% were not sure about it. Several other studies have shown that robotic surgery is not well received by patients compared to conventional surgery. A study found that 33.7% of patients' willingness to undergo robotic surgery was low [1]. In another study, robotic surgery was preferred by 6.4% of patients undergoing pelvic organ prolapse surgery [21]. In the Singapore study, 79% of the participants were concerned about internal injury by the robot, and 55.1% thought the robot would perform a wrong procedure [1]. Similarly, in our study, the fear of internal injury caused by the robot was 58.3% of those who declined robotic surgery. Hence, patients should be educated regarding robotics since it is evident that it has lower chances of causing perioperative complications than abdominal open or laparoscopic surgery [22]. In another study, 50% of participants thought that robotic surgeons are more skilled or competent than non-robotic surgeons. In our study, 35.5% of respondents believed that surgeons trained in robotic surgery were more skilled [23]. The findings of our study estimated that 48% of the respondents thought robotic surgery cost was higher, which could have contributed to the decline of the procedure. Based on another study, 44.9% of participants thought robotic surgery would be costly, and 63.7% stated they would not prefer it [1]. Due to its significantly higher cost than its laparoscopic counterpart, robotic surgery has been a controversial topic. It costs approximately £1300 per robotic prostatectomy due to the use of disposable instruments. The system's software costs around £1.5 million [24]. All these costs will affect patients and affect their opinion on robotic surgery. Despite the high costs, few randomized studies have shown robotic surgery to have advantages over conventional procedures [25]. Regarding the perception of hospitals and robotics, 61.8% of the patients perceived hospitals employing RS as better than hospitals that do not. However, in the Kai et al. study, 53.6% of the patients viewed hospitals using RS as similar to those that do not use it [1].

In our study, 34.9% of the respondents and 67.2% of the participants who preferred robotic surgery believed it may eventually replace the present practice. When we compare it to the study by Boys et al., only 45% of their participants preferred robotic surgery over conventional minimally invasive techniques if the surgery was necessary and could be performed by a robot [16, 26]. Similarly to a study in Kuwait, 35.4% of respondents said they would choose robotic surgery over conventional surgery [26]. In our study, 38.2% of respondents and 78.1% of those who preferred robotic surgery believed that using robotic surgery could lead to better surgical outcomes. In the Singapore study, 29% thought robotic surgery could offer better outcomes [1]. However, the majority of the participants in the Boys et al. study (72%) believed that robotic surgeries had better outcomes. This might be explained by the fact that the Boys study included healthcare workers in their survey, which makes them more informed about robotic surgery as they work in the healthcare field [16].

Limitations

One of the limitations of our study is that our participants were relatively young and well-educated. Also, Berkson's bias is possible because of our convenient sampling method. We only included patients who attended the OBGYN, thoracic, colorectal, and urology clinics. This may not be representative of the general population. In addition, our survey was not validated for our population but was adopted from a pre-existing survey on the same topic over a larger population. Also, our study may not be generalized as it was conducted in a single center in Riyadh, Saudi Arabia. Finally, our study was conducted in an urban area where higher education is available and information acquisition is easier. The results might have changed if rural areas were involved.

Recommendations

We assessed patients' knowledge, awareness, attitude, and future expectations of attending surgical outpatient clinics. Yet, it is evident from the results of this study that patients lack information regarding robotic surgery in all aspects of our assessment. Even though robotics in surgery has increased over the years, patients still seem to understand it differentiates it from other procedures. Patients, their friends, and loved ones must grasp the operation they will undertake. Thus, we recommend more comprehensive education regarding robotics in the field of surgery and for more research to be conducted. Furthermore, healthcare workers should also be examined with a similar assessment.

## Conclusions

Our study concluded that there is a lack of awareness about robotic surgeries, and most participants' information was from social media. In addition, most participants did not favor robotic surgeries or were unsure of them. Also, most of them believed that hospitals utilizing robotic surgeries are better. Participants were most worried about faults that can cause internal injuries.
